# Differential cognitive impairment for diverse forms of multiple sclerosis

**DOI:** 10.1186/1471-2202-7-39

**Published:** 2006-05-19

**Authors:** Javier J Gonzalez-Rosa, Manuel Vazquez-Marrufo, Encarnacion Vaquero, Pablo Duque, Monica Borges, Miguel A Gamero, Carlos M Gomez, Guillermo Izquierdo

**Affiliations:** 1 Multiple Sclerosis Unit, Virgen Macarena Hospital, Avda Dr Fedriani s/n, 41009 Seville, Spain; 2 Laboratory of Psychophysiology, Departament of Experimental Psychology, University of Seville, Camilo Jose Cela s/n, 41018 Seville, Spain

## Abstract

**Background:**

Cognitive impairment is a common feature in multiple sclerosis (MS) patients and occurs in 60% of all cases. Unfortunately, neurological examination does not always agree with the neuropsychological evaluation in determining the cognitive profile of the patient. On the other hand, psychophysiological techniques such as event-related potentials (ERPs) can help in evaluating cognitive impairment in different pathologies.

Behavioural responses and EEG signals were recorded during the experiment in three experimental groups: 1) a relapsing-remitting group (RRMS), 2) a benign multiple sclerosis group (BMS) and 3) a Control group. The paradigm employed was a spatial attention task with central cues (Posner experiment). The main aim was to observe the differences in the performance (behavioural variables) and in the latency and amplitude of the ERP components among these groups.

**Results:**

Our data indicate that both MS groups showed poorer task performance (longer reaction times and lower percentage of correct responses), a latency delay for the N1 and P300 component, and a different amplitude for the frontal N1. Moreover, the deficit in the BMS group, indexed by behavioural and pyschophysiological variables, was more pronounced compared to the RRMS group.

**Conclusion:**

The present results suggest a cognitive impairment in the information processing in all of these patients. Comparing both pathological groups, cognitive impairment was more accentuated in the BMS group compared to the RMSS group. This suggests a silent deterioration of cognitive skills for the BMS that is not usually treated with pharmacological or neuropsychological therapy.

## Background

Multiple sclerosis (MS) is a neurodegenerative disease of the Central Nervous System (CNS) in which early symptoms can appear in young adults between 20 and 40 years old. This condition damages the myelin shield of neurons in the brain, spinal cord and optical nerves. As a consequence of that, it produces sensory, motor and cognitive impairments that produce physical disabilities [1,2].

Most MS patients present a clinical course in which exacerbations and remissions occur (85–90%). If this pattern is established, MS is classified as MS relapsing-remitting form (RRMS). After 10 years suffering this form of the disease, 50% of patients enter in a progressive phase of the illness defined as Secondary Progressive. The Primary Progressive form is characterized by a continuous progression from the very beginning, without signs of remission. A reduced number of patients present a progressive clinical course and some of them can present bursts (Progressive-Relapsing form). Finally, a 20% of patients show a benign course (BMS) of the clinical condition (see [1-6] for reviews).

The cognitive deterioration is highly prevalent as one of the symptoms accompanying the general deterioration of patients [7-9]. The more common functions usually impaired are sustained attention, speed processing, abstract reasoning, verbal fluency and visuo-spatial perception among others. The cognitive deterioration pattern must be somehow associated with the anatomopathology, number and location of lesions, producing then a high individual variability. However, it is difficult to directly associate the neurological and neuropsychological results [10,11], and the lack of correspondence between Magnetic Resonance Imaging (MRI) characterised lesions and cognitive deterioration occurs very frequently.

This discrepancy between cognitive-behavioural functioning and MRI signs has promoted the use of other techniques from neuroscience disciplines to objectively explore the relation between brain dysfunctions and neuropsychological deterioration. One of these techniques is the Event-Related-Potentials (ERPs). The endogenous components of ERPs are particularly linked to the cognitive associated symptoms of the MS condition [12,13].

The reliability of ERPs results is in the same order to those obtained in laboratory tests [14]. Moreover, the temporal resolution of this technique is much better than other metabolic techniques as positron emission tomography or functional MRI. The P300 component is being used regularly in clinical practice [14]. Latency and amplitude of P300 are sensitive to neural degenerative conditions as several types of dementias and psychiatric diseases [15-19]. The current analytical tools applied to the electroencephalogram (EEG) allow to temporally and spatially localize certain phases of cognitive processing [20]. EEG techniques as ERPs, quantitative spectral EEG and coherence analysis have shown that the EEG is modified in MS with respect to controls [21-25]. The combination of EEG recording with an experimental design that allows measuring cognitive function is a powerful method to evaluate cognitive function in controls and patients. In order to study attentional mechanisms, the so-called Posner paradigm with central cues has been broadly used [26,27].

In this test, the subject must indicate the location of a target stimulus. The location of the target stimuli is previously cued (validly or invalidly) by a central arrow. The behavioural measures are response times, and percentage of correct responses, or response errors. There is a benefit in the RTs if the target stimulus appears in the cued location. On the contrary, there is a cost in the RTs if the target appears in the uncued location.

The results in this paradigm and others have allowed to propose a theoretical framework about different attentional operations: 1) "disengagement" from the last attentional focus 2) "moving" the attention to the new location 3) "engagement" to the new location [28]. The robustness of the model has also been tested by different studies including neuropsychological testing in injured patients, animal models, neuroimaging and behavioural variables. All these studies have suggested an attentional neural network including fronto-parietal areas, superior colliculus and pulvinar nucleus [28]. Moreover, the central cue is also able to prepare the specific sensory and motor circuits that are going to be needed to complete the task upon the available information provided by the central cue[29,30].

The aim of present study was to understand if attentional mechanism, as measured in the central cue Posner's paradigm (spatial attention and stimuli discrimination), is impaired in MS patients. Our hypothesis is that given the distributed nature of the attentional network, the demyelinization process would affect its function in the different brain areas of the RRMS patients. Furthermore, the possibility of testing a group of patients presenting BMS form would allow to establish the possible cognitive deterioration of these patients, which is not always detected by neurological and neuropsychological examination, as it has been suggested by other authors [31]. The analysis of ERPs component in both groups of MS patients would help in the definition of the possible cognitive deterioration of BMS patients.

## Results

**Figure 1:**
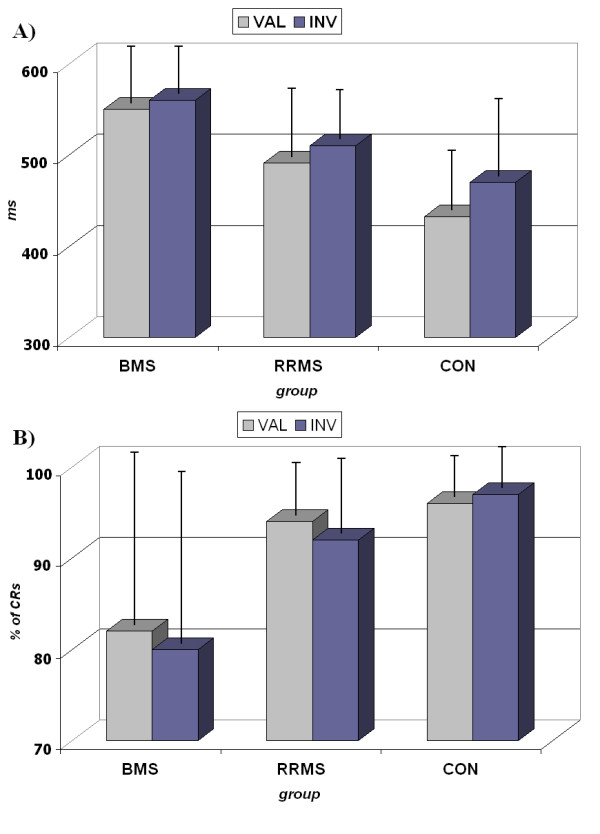
Behavioural results. **2A**. Response times (RTs) for valid and invalid cues. Notice (i) the shorter RTs in the valid cue with respect to invalid cues (except in the BMS group) and, (ii) the shorter RTs in the Control group with respect to the MS groups **2B**. Percentage of Correct Responses (CRs). Notice the low number of hits in the BMS group. CON: Control Group; RRMS: relapsing-remitting group; BMS: benign group.

The RTs of the valid cue condition were statistically significantly shorter compared to the invalid ones, showing a facilitated visuo-motor processing when attention is directed to the validly cued position (F [1, 42] = 37.148, p < 0.001). This facilitation was present for the Control and the RRMS group (F [2, 42] = 4.450, p < 0.018), although the Control group shows a higher difference between those conditions (34 ms) than the RRMS group (18 ms). The BMS group, however, did not show statistically significant differences between valid and invalid reaction times (see Figure 1 and Table 1).

The intersubject comparison showed that there was an effect of the group (F [2, 42] = 14.947, p < 0.001). The post-hoc analysis showed that the Control group responded faster than the RRMS group (p < 0.013) and the BMS group (p < 0.001). Regarding the comparison between RRMS and BMS groups, RRMS patients were statistically significantly faster in responding to targets than the BMS group (p < 0.021) (see Figure 1 and Table 1).

In the inter-subject comparison, the analysis of the percentage of correct responses showed that there was an effect of the group (F [2, 42] = 6.790, p < 0.003). The post-hoc analysis revealed that this difference was statistically significant in the comparison between the Control group and the BMS group (p < 0.002) and between the RRMS and BMS groups (p < 0.022), and was not statistically significant between the RRMS group and the Control (see Figure 1 and Table 1).

With regard to the analysis of errors, the ANOVA showed a statistically significant interaction between the effects of the following factors: cue, type of error and group (F [2, 42] = 4.136; p < 0.020). The interpretation of this result comes from different evidences. First of all, there were more errors in the valid cue than in the invalid condition, and the most frequent type of error was the one termed "missed". Moreover, this difference was exclusively present for the BMS group compared to RRMS (p < 0.033) and Control groups (p < 0.003).

In the analysis of the amplitude of the P1 component, there was not statistical difference between the different groups (F [2, 42] = 1.786; p < 0.180). However, the observed P1 amplitude in the Control and RRMS groups showed higher amplitude for valid trials compared to invalid trials, which was not observed in the BMS group.

**Figure 2:**
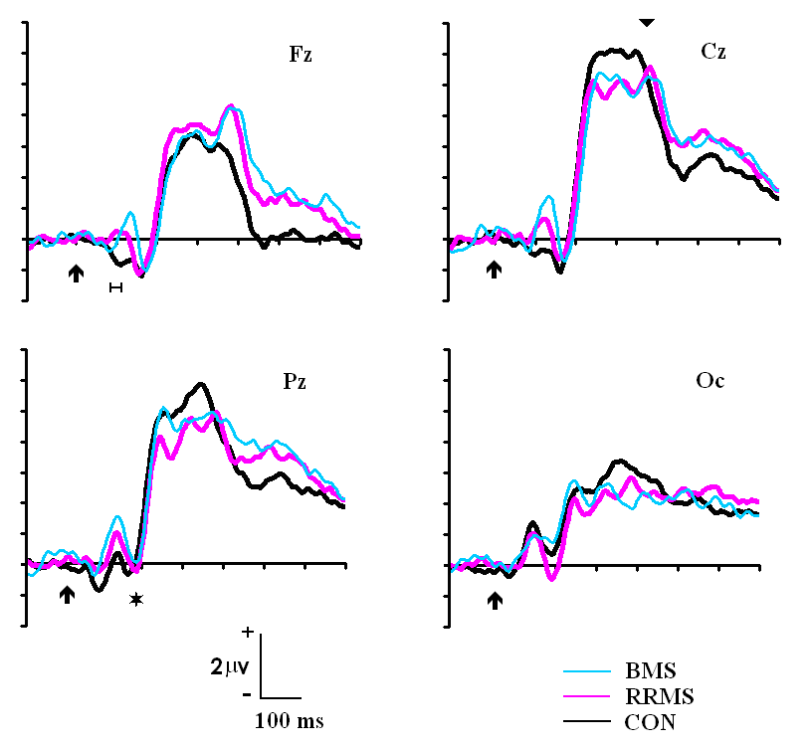
ERPs to the standard stimuli in the Fz, Cz, Pz and Oc electrodes for the Control, RRMS and BMS groups. Notice the delayed latency of the P300 (inverted triangle) and posterior N1 (asterisk). Also notice the lack of the early frontal N1 in the BMS and RRMS group (the horizontal line indicates the time window used for statistical analysis). CON: Control group; RRMS: relapsing-remitting group; BMS: benign group

There was no statistical difference for the P1 latency between the different groups. With respect to the latency of the N1 component, the ANOVA showed a statistically significant effect of the electrode factor (F [3, 42] = 30.226, p < 0.001). The post-hoc analysis showed that the differences were due to anterior-posterior differences in latencies (Fz-Oc = p < 0.001; Fz-Pz = p < 0.004; Oc-Cz = p < 0.001). These differences appeared in both: valid and invalid. The effect of the group factor was statistically significant for the latency of the N1 component (F [2, 42] = 4.259, p < 0.021). The post-hoc analysis revealed that this difference was due to a statistically significant delayed latency of the N1 component of the BMS group with respect to the Control group (p < 0.017) (see Figure 2 and Table 1).

For the latency of the P300 component, the ANOVA analysis revealed a statistically significant effect of the group factor (F [2, 42] = 24.879, p < 0.001). The post-hoc analysis showed that the latency for the Control group (332 ± 23 ms) was faster (p < 0.001) than the latency for both MS groups (RRMS: 369 ± 28 ms; BMS: 388 ± 38 ms). The post-hoc analysis also confirmed the statistically signficant differences between MS groups (p < 0.042).

The amplitude of the early frontal N1 was clearly different between the Control group and the MS groups. In fact, the MS groups presented, at that latency, a positive voltage in the Fz electrode instead of the negativity observed in controls (Figure 2). The amplitude of the early N1 component at Fz electrode showed a statistical difference for the effect of the group factor [F (1, 42) = 6.585; p < 0.003]. The post-hoc analysis showed a statistical difference between Control and RRMS groups (p < 0.036) and between Control compared to BMS group (p < 0.005). There were not statistically significant differences between RRMS and BMS groups.

## Discussion

The performance in the attentional task was poorer for BMS group compared to the RRMS group and for both MS groups with respect to the Control group. This difference in behaviour was statistically significant and was consistent across all behavioural variables (RTs, errors and percentage of correct responses). This difference cannot be explained by a speed-accuracy trade-off because MS groups were also less accurate than Control group, particularly in the case of BMS group. The increase in RTs is a common result obtained in the MS literature [24,32] and suggests that BMS patients also show a deterioration in visuo-motor processing as other subtypes of MS.

The behavioural results, using the central cue paradigm, replicate the studies where an improvement of the RTs is observed for the valid cues compared to the invalid ones [26,33]. This is explained by the need of a reorienting mechanism in the invalid condition that it is not necessary for the valid condition. The differences obtained in the RTs during the Posner paradigm with central cues indicate that the performance in the spatial attention processing in the BMS group is impaired, given that there was not a statistically significant difference between RTs for valid and invalid cues in the BMS group and the latencies were delayed compared to the Control group. This impairment could involve both orienting (indicated by the longer RTs in both conditions for BMS) and reorienting mechanisms (indicated by the lack of statistical significance between valid and invalid conditions).

The present study reveals that validity effects were obtained in Control and RRMS groups but were not obtained in the BMS group. It is possible that the lack of the validity effect in the BMS group is caused by a prolonged duration of the moving and engaging operations, which would produce a divided attention situation instead of an oriented spatial attention condition, as it occurs in the Control and RRMS groups.

To check if these patients were orienting or not to the cue, we checked if the P1 component showed a change of amplitude to the stimuli when they were presented in the validly field with respect to when the cue invalidly cued the position of the stimulus [34]. The result of this analysis was that although there were not statistically significant differences, a higher amplitude was observed for the validly cued stimulus with respect to the invalidly cued stimulus in the Control and RRMS groups. However, for the BMS group, the amplitude of P1 was practically equal for both conditions, suggesting that it is probable that these patients carried out a divided attention setting during the course of the task.

The analysis of errors yielded a similar conclusion than the RTs analysis, an increase in the number of errors in the BMS group with respect to RRMS and controls. The overall conclusion is that both MS groups showed a behavioural deterioration compared to controls, but this deterioration was higher in the BMS group than in the RRMS group.

The latency of the P1 component did not reflect statistically significant differences among the experimental groups. These results suggest that the differences found in the behavioural variables among the diverse groups are not due to a different processing in the speed of sensory-visual pathways. One interesting phenomenon appearing in the ERPs at the same latency than P1 was the inversion of polarity that occurs in MS patients of the early N1 frontal component. The early N1 frontal component that appeared in controls has been previously described [35]. They have suggested that this initial volley of information arrives to the frontal cortex through the dorsal stream and can be used for an alerting function in the frontal cortex while more elaborated information arrives from computation in higher order visual cortex. The patient's inversion of polarity obtained in the N1 frontal component could be due to a lack of this communication because of central demyelinization. If the frontal N1 component is not formed, the posterior P1 could have a much more frontal extension and produce the inversion of polarity observed in MS patients in frontal electrodes. A functional consequence of that would be the lack of the fronto-occipital attentional control that would occur after the frontal N1 component [35].

The latency of the posterior N1 was increased in the BMS group with respect to controls. Given the implication of the N1 in the object discrimination process and its localization in the occipito-ventral areas [20,29], the increase of N1 latency in BMS group would implicate a delay in visual discrimination processes with respect to controls.

On the other hand, we can observe the significant delay in the latency of the P300 component for MS groups, being the BMS group the one that exhibited the longest latencies when compared to the Control group. Indeed, the BMS group showed statistical differences even with the RRMS group. Part of this delay is partially due to the N1 latency delay, at least for the BMS group. This latency delay of the P3 component has been observed in previous studies with different pathologies. In the particular case of the multiple sclerosis, Polich [23] suggested a cognitive deficit associated to the anomalies in the latency and amplitude of the P300 component. Ellger [25] presented a significant delay of the latency of the P300 in different groups of MS, although the BMS was not studied. Aminof [36] indicated that the changes in the components of the ERPs in the MS are due to a central disorder that correlates with the patient's cognitive state. Dujardin [37] suggested, using behavioural variables, that the attentional deficit in MS does not depend on the attentional gain mechanisms. Instead, the deficit would be caused by the lack of central resources in the processing of visual information. Our study reinforces these previous empirical results and supports the hypothesis that the deficit exhibited is related to the cognitive processing and not only dependent on sensory processing deterioration.

It is necessary to highlight that in our study, the measures of P300 peak latency were carried out with standard stimuli (that does not require motor response). Therefore, it is possible to discard any contribution from motor potentials. Moreover, the impaired performance in behaviour for the MS patients observed in this study cannot be explained exclusively in terms of motor dysfunction. The delay in the P300 component has to be related to a cognitive impairment in the MS patients. It is important to highlight that the capacity of attentional orientation in the visual world is a function of enormous importance. It is not only related to the visuo-attentional process itself, but it is involved in many processes (e.g.: learning, memory, etc).

As mentioned above, one of the most surprising results is the worse execution in the task for what is called benign course of disease (BMS group). In the clinical practice, these patients show a slow evolution of the disease without the presence of evident clinical signs for many years and, therefore, with no pharmacological therapy applied [1-4,38-40]. However, our test has allowed us to check that these patients show a poorer behavioural execution in terms of RTs and errors compared with the RRMS group. Moreover, the BMS group presented the most impaired ERPs in both the latencies of the posterior N1 and P300, and in the absence of the frontal N1.

According to those results, it is possible to ask if these techniques could be useful for diagnostic purposes. The present results suggest indeed that differences take place among the different studied groups. However, given the number of cases studied in our study, it seems daring to define these measures as diagnostic tools. We believe that more studies are necessary to confirm the validity of these parameters in the diagnosis of multiple sclerosis and be included in future diagnostic criteria.

**Figure 3:**
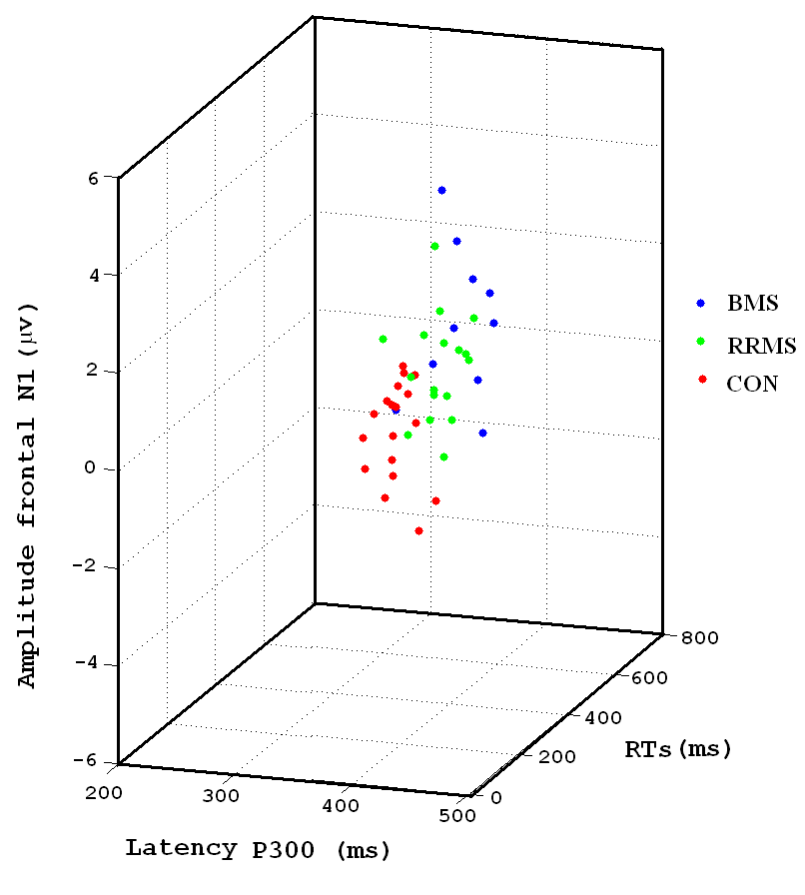
3D scatter-plot that represents RTs, P300 latency and amplitude of the N1 frontal for the different groups. Notice that each group occupies a region in the 3D space.

However, the evidences found in the study suggest that the behavioural and psychophysiological measures can be good estimators of the degree of attentional impairment in multiple sclerosis and help professionals to apply the appropriate therapeutic measures (see Figure 3).

On the other hand, these results prove that the neurological and cognitive evolution of a MS patient does not correlate. In fact, in subjects with a low level of neurological incapacity (EDSS < 3 or 4), the cognitive profile is affected. To conclude, these data suggest that the cognitive impairment in MS could develop in a subtle way during the course of the disease, producing a deterioration similar to that experienced by RRMS patients. This fact points to the necessity of increasing the research in clinical and laboratory parameters in the prediction and more proper definition of benign form of multiple sclerosis [31]. These results confirm that the use of psychophysiological techniques would be recommended to explore the cognitive profile in multiple sclerosis patients as a complementary test for the neurological and neuropsychological examination.

## Conclusion

Cognitive deterioration is a highly prevalent symptom accompanying the general deterioration of MS patients. In the present experiment, these patients showed an attentional impairment indexed by ERPs and behavioural responses. The main result was that BMS group presented a higher deterioration than RRMS group. The latter result suggests that it could be necessary to follow up the cognitive evolution of the patients with BMS and to consider therapeutic alternatives to those offered today. Psychophysiological techniques combined with specific tasks (e.g. Posner paradigm) can be helpful in evaluating cognitive impairment in MS.

## Methods

### Subjects and procedure

Three different groups of subjects participated in the study: The first group included 17 patients (women 12; age 38.88 ± 9.04 years old) diagnosed with RRMS without clear signs of motor impairment, and with a disease duration of 4.67 ± 4.13 years. The score in Multiple Sclerosis Expanded Disability Status Scale (EDSS) was 1.6 ± 0.9. The second group included 10 patients (women 6; age 42.30 ± 7.21 years old) diagnosed with BMS. These patients had a disease duration of 12.09 ± 4.77 years and at least 8 years of disease evolution scoring under 3.5 in the EDSS scale. Diagnosis was made following the criteria of Poser [1]. The exclusion criteria for the MS groups were: the presence of a relapse, medication in the last month, and/or the presence of clear signs of depression. All patients were clinically stable at baseline (i.e., had no exacerbations within several months before of study entry). There were no patients treated with any medication at the time of evaluation, and only a few presented history of corticoids treatment in past relapses. Patients previously assessed at the Multiple Sclerosis Unit of the Neurology service of the Virgen Macarena Hospital (Seville, Spain) participated voluntarily in the Psychophysiological testing.

The data from both clinical groups were compared with a group of 18 healthy subjects (women 15; age 36.54 ± 8.73 years old) similar in age, gender proportion and educational level. The experimental protocol was approved by the ethical committee of the Hospital. After a full explanation of the nature and general objectives of the experiments, a written consent statement was obtained from subjects: MS patients and controls.

### Experimental protocol

**Figure 4:**
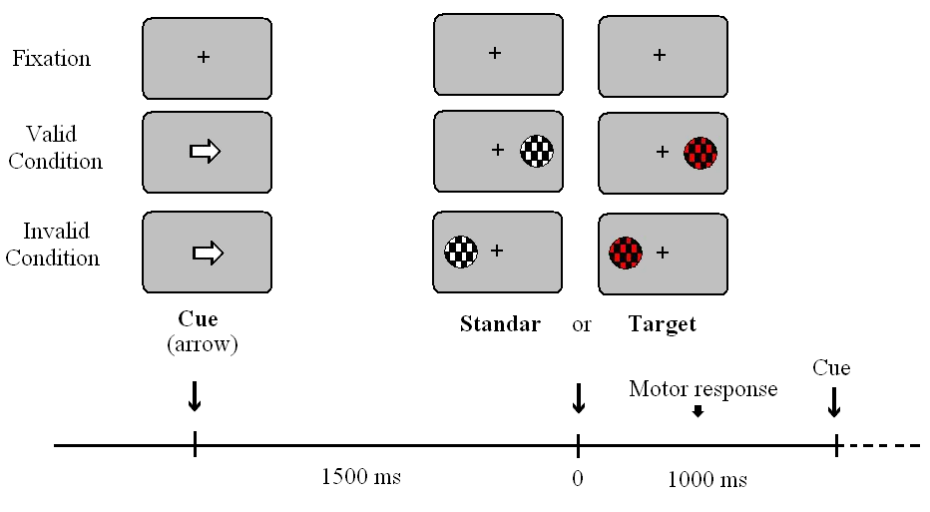
Experimental paradigm.

Behavioural responses were recorded during the Posner paradigm. Five blocks with 200 trials each were presented. The subjects had 2–3 minutes of rest between blocks. A trial consisted in a central cue (lasting 300 ms) pointing to the left or to the right side of the screen, where a target (mandatory response) or a standard stimulus (no response) appears. Targets and standards also lasted 300 ms. Total time for the trial was 1.5 sec and the inter-trial time was 1 sec. The cue (a white arrow) appeared at the screen centre. In standards and targets trials the cue could point to the position where the stimulus appears (valid trials, 80%) or to the opposite side (invalid trials, 20%) (see Figure 4). The shape of the target stimuli appearing randomly at left or right side was a circle with a pattern of black and red checkerboard subtending a visual angle of 2.46 degrees. The standards presented the same shape than targets but the colours were changed to black and white. Subjects had to press the left button of the mouse with their dominant hand when targets appeared in the left side, and the right button when the target appeared in the right side. Therefore, left valid, left invalid, right valid and right invalid trials appeared for both: standard and target trials. We decided to use a modified version of the Posner's paradigm with central cues in which a 75% of standard stimuli appeared. We chose that proportion in order to be able to evaluate enough behavioural responses in the 25 % of target trials, but at the same time, to be able to analyse cognitive ERPs without motor contamination during the 75% of standard trials.

The EEG was recorded from 13 electrodes (Fz, Cz, Pz, F3, F4, C3, F4, P3, F4, T5, F6, O1, O2) from the 10–20 International System [41]. The electrodes were referenced to the left mastoid and re-referenced off-line to the linked mastoid. Data were filtered using a band-pass of 0.01–100 Hz (1/2 amplitude low- and high-frequency cut-offs); the amplification gain was 20,000. The EOG was recorded with bipolar recording by means of electrodes situated in the external canthi of the ocular orbits and in the inferior and superior positions of the left orbit. The impedance was kept under 5 kΩ. EEG was digitised at a frequency rate of 500 Hz. Artifacts were automatically detected and visually revised. The trials in which HEOG artefacts higher than ± 70 μV were detected at electrodes Fz, Cz or Pz, were rejected. The baseline was 100 ms prior to thestandard and target stimuli. The different experimental conditions were averaged independently.

### Statistical analysis

The behavioural parameters analysed were Reaction Times (RTs) to the target stimuli, percentage of correct responses (CRs) and errors. The considered errors were: *missed *when no response to the target occurred, or the response occurred 700 ms after target stimulus; *target error *when responses to the targets occurred with the wrong finger; and *false positive *when the response was to standard stimuli. An ANOVA design for repeated measurements was used to analyse the RTs and CRs data. The intra-subject factor was the validity of the cue (two levels: valid vs. invalid) and the intersubject factor was the subject's group (three levels: RRMS, BMS and Controls). The Bonferroni correction was used for post-hoc analysis. In the case of errors, a mixed factorial ANOVA designed for repeated measurements was used. The intra-subject factors were: the validity of the cue (two levels: valid vs. Invalid) and the classification of errors (misses, target error, and false positive); the intersubject factor was the subject's group (three levels: RRMS, BMS and Controls).

Latency was measured for P1, N1 and P300 components. The P1 latency was obtained as the highest positive deflection in the time window 90–160 ms for the electrode Oc (electrode Oc was computed off-line as the mean voltage of O1 and O2); the N1 component as the highest negative deflection in the time window 130–210 ms for the electrodes Fz, Cz, Pz and Oc, and the P300 as the highest positive deflection in the time window 280–400 ms for the electrodes Fz, Cz and Pz.

The statistical significance of latency differences in the P1 component was tested independently by means of one-factor ANOVA (three levels: RRMS, BMS and Controls). To analyse the latency of the N1 and P300 component, a mixed factorial designed for repeated measurements was applied to the latency data. For N1 and P300 analysis, the intra-subject factors were: a) validity of the cue (two levels: Valid and invalid) and b) electrode. The inter-subject factor was the subject's group (Three levels: RRMS, BMS and Controls).

Our initial objectives were related to the latency analysis of ERPs. However, after visual inspection of ERPs in the midline, an inversion of polarity in the frontal N1 component was obvious for the MS groups (see Figure 2). This highly unexpected result motivated the amplitude analysis of the frontal N1 in the Fz electrode in which a positive deflection appeared for the MS groups. The mean voltage between 110 and 130 ms in the electrode Fz was computed in the three subject groups. An ANOVA design for repeated measurements was used to analyse the amplitude of the frontal N1. The intra-subject factor was the validity of the cue (two levels: valid vs. Invalid), and the intersubject factor was the subject's group (three levels: RRMS, BMS and Controls).

In order to verify the effect of validity in the early phase of attentional processing, the differences of amplitude for the P1 component were analysed. The P1 amplitude was calculated based in a positive deflection which appeared in the posterior electrodes in all groups. The mean voltage occurring between 120 and 150 ms in the electrode P3 and P4 was computed in all stimular conditions (left valid, left invalid, right valid and right invalid trials). An ANOVA design for repeated measurements was used to analyse the amplitude of the P1. The intra-subject factors were: a) validity of the cue (two levels: valid vs. invalid), b) side of visual field stimulation (two levels: left vs. right) and c) electrode (two levels: p3 and p4). The intersubject factor was the subject's group (three levels: RRMS, BMS and Controls).

## Abbreviations

ANOVA analyses of variance

BMS benign multiple sclerosis

CRs correct responses

EDSS expanded disability status scale

EEG electroencephalography

ERPs event-related potentials

MRI Magnetic Resonance Imaging

MS multiple sclerosis

RRMS relapsing-remitting multiple sclerosis

RTs reaction times

## Authors' contributions

MV and JJGR, with CG and PD participated in the planning of the study. JJGR and MV participated in the acquisition and execution of the study and performed the data analysis. MV, CG, GI, EV, PD and JJGR contributed to the interpretation of results. The patients were selected by PD, MB, MAG and GI. JJGR drafted the manuscript. The manuscript was subsequently revised by MV, CG, EV and PD and GI, and all authors gave final approval.

## Figures and Tables

**Table 1:** Descriptive and Statistical results of the behavioural and ERPs data. CON: Control Group; RRMS: relapsing-remitting group; BMS: benign group.

	Reaction Times			Correct Response %			Latency N1			Latency P300			Amplitude N1 frontal		
***Descriptive***	*Valid*	*Invalid*	*Total*	*Valid*	*Invalid*	*Total*	*Valid*	*Invalid*	*Total*	*Valid*	*Invalid*	*Total*	*Valid*	*Invalid*	*Total*
BMS	**549 **± 63	**560 **± 45	**555 **± 51	**81 **± 23	**79 **± 21	**80 **± 23	**179 **± 14	**179 **± 10	**179 **± 12	**383 **± 22	**386 **± 21	**385 **± 21	**.97 **± 1.2	**.97 **± 2.1	**.97 **± 1.6
RRMS	**492 **± 47	**510 **± 43	**501 **± 43	**94 **± 05	**92 **± 07	**93 **± 06	**172 **± 13	**170 **± 15	**171 **± 14	**365 **± 19	**372 **± 24	**368 ± **21	**.47 **± .1.2	**.33 **± 1.5	**.40 **± 1.3
CON	**433 **± 50	**467 **± 56	**450 ± **53	**96 **± 04	**97 **± 05	**96 **± 05	**164 **± 12	**167 **± 11	**165 **± 12	**341 **± 15	**342 **± 12	**341 **± 14	**-.85 **± 2.4	**-.63 **± .87	**-.74 **± 1.6

***Anova***	*F*		*p*	*F*		*p*	*F*		*p*	*F*		*p*	*F*		*p*
Cue	37.148		**<0.001**	0.785		0.381	0.001		0.992	1.376		0.258	0.009		0.925
Elec							30.226		**<0.001**	3.731		**0.017**			
Cue*Group	4.450		**0.018**	0.575		0.567	0.975		0.386	0.398		0.674	0.151		0.861
Group	14.947		**<0.001**	6.790		**0.003**	4.259		**0.021**	24.879		**<0.001**	6.685		**0.003**

***Post Hoc***	*RRMS*	*BMS*	*CON*	*RRMS*	*BMS*	*CON*	*RRMS*	*BMS*	*CON*	*RRMS*	*BMS*	*CON*	*RRMS*	*BMS*	*CON*
*RRMS*		**0.021**	**0.013**		**0.022**	0.980		0.287	0.517		**0.042**	**<0.001**		0.827	**0.036**
*BMS*			**<0.001**			**0.002**			**0.017**			**<0.001**			**0.005**
